# Efficient generation of stable, heritable gene edits in wheat using CRISPR/Cas9

**DOI:** 10.1186/s12870-018-1433-z

**Published:** 2018-10-03

**Authors:** Rhian M Howells, Melanie Craze, Sarah Bowden, Emma J Wallington

**Affiliations:** The John Bingham Laboratory, NIAB, Huntingdon Road, Cambridge, CB3 0LE UK

**Keywords:** *Triticum aestivum*, *Hordeum vulgare*, CRISPR\Cas9, Genome editing, Heritability, Knockout

## Abstract

**Background:**

The use of CRISPR/Cas9 systems could prove to be a valuable tool in crop research, providing the ability to fully knockout gene function in complex genomes or to precisely adjust gene function by knockout of individual alleles.

**Results:**

We compare gene editing in hexaploid wheat (*Triticum aestivum*) with diploid barley (*Hordeum vulgare*), using a combination of single genome and tri-genome targeting. High efficiency gene editing, 11–17% for single genome targeted guides and 5% for tri-genome targeted guides, was achieved in wheat using stable *Agrobacterium*-mediated transformation. Gene editing in wheat was shown to be predominantly heterozygous, edits were inherited in a Mendelian fashion over multiple generations and no off-target effects were observed. Comparison of editing between the two species demonstrated that more stable, heritable edits were produced in wheat, whilst barley exhibited continued and somatic editing.

**Conclusion:**

Our work shows the potential to obtain stable edited transgene-free wheat lines in 36 weeks through only two generations and that targeted mutagenesis of individual homeologues within the wheat genome is achievable with a modest amount of effort, and without off-target mutations or the need for lengthy crossing strategies.

**Electronic supplementary material:**

The online version of this article (10.1186/s12870-018-1433-z) contains supplementary material, which is available to authorized users.

## Background

The ability to examine valuable agronomic traits within crop species has, until recently, been possible only by extensive crossing programmes or the use of genetic modification to insert or silence target genes. The use of RNAi silencing in complex polyploid species mostly results in reduced expression rather than complete knockout, making phenotypic evaluation difficult [1]. Recent developments in the area of gene editing could, therefore, be invaluable as a means of reliably producing true knockouts, particularly in polyploid species.

The use of CRISPR (clustered regularly interspaced short palindromic repeats)/Cas9 systems is increasingly reported in plant species. The system requires two components to be expressed within the same plant cell, the Cas9 adapted from *Streptococcus pyogenes* and a short guide RNA (gRNA) which targets the Cas9 to the required genomic sequence. Despite the progress made in plants, gene editing remains challenging in transformation-recalcitrant species, with relatively few experiments reported in the major crop species. Within the monocots, editing has been reported in maize, rice and barley [2–4]; however, questions remain from these studies regarding both the editing efficiency and heritability of the induced edits [5, 6].

In bread wheat (*Triticum aestivum*), editing has been reported in protoplast systems [7, 8] and, more recently, edited wheat plants have been regenerated after transient expression of Cas9 and guides introduced via particle bombardment [9–11]. However, both the design of the gRNA and the downstream analysis of potential edits are more difficult due to the complex hexaploid nature of the wheat genome. Only limited publications exist on the production of full grown stable edited wheat plants, rather than protoplast systems and all have used a biolistic approach. This method often results in very high copy number complex insertions at multiple loci, as a consequence it can be difficult to remove the Cas9/guide cassette in subsequent generations, whilst also segregating the possible bi-allellic edits in a hexaploid genome. Here we present the first example of gene editing in wheat using *Agrobacterium*-mediated transformation.

To facilitate identification of homeologue specific gene knockouts we have targeted phytoene desaturase (PDS) which is present as a single gene in the wheat genome. PDS is a carotenoid pathway enzyme, which performs the desaturation of phytoene to zeta-carotene [12]. The reduction or loss of function of PDS has been shown to result in a photobleaching phenotype and has been widely used in plants as a visual screen for gene knockout. Virus-induced gene silencing (VIGS; [13]) and gene silencing via RNAi [14] have both been demonstrated using wheat PDS (*TaPDS*). The conserved nature of PDS between species and the potential to visually phenotype knockout lines make it an ideal candidate gene. In this study we employ it to analyse both the ability to edit genes, and to compare the efficiency and characteristics of the gene editing cassette components in wheat and barley.

The complexity of wheat has meant that genes and regulatory sequences transferred from other, even monocot, species function differently and, therefore, constructs and new technologies can require a greater degree of optimisation. Accordingly, we designed a suite of constructs that enabled testing of different cassette components, including Pol III promoters and gRNA scaffolds in order to progress towards the optimum vector design for gene editing in wheat. Here we demonstrate the production of edited wheat plants using *Agrobacterium-*mediated transformation. Edits have been produced at a high efficiency without off-target effects and have been shown to be stably inherited through multiple generations to produce lines without the T-DNA bearing the Cas9 and guide cassette sequences.

## Results

Our target gene, *TaPDS*, is present in wheat as a single copy on each of the three constituent genomes. To exclude any potential for varietal SNPs which might affect gene editing, the first five exons and four introns of all three homoeologues were cloned and sequenced from a US spring wheat variety, Fielder, which is the preferred cultivar for efficient transformation. Comparison of the resulting genomic sequences from Fielder (Additional file 1: Figure S1) showed that the percentage identity of this portion of *TaPDS* between the three homoeologues was 95–96%. Sufficient differences were identified within the introns of *TaPDS* homoeologues to enable design of genome specific primers for PCR amplification (Additional file 2: Table S1), while exonic similarity was sufficient to allow gRNA sequences to be identified for both genome-specific edits and to target all three homoeologues simultaneously with a guide of 100% match (Additional file 1: Figure S1). The DNA sequence similarity of *TaPDS* to the orthologous gene in barley (*Hordeum vulgare,* cv. Golden Promise) was such that, in some cases, the same gRNA could be used for editing in both species. This ability to use the same construct within both to target PDS would not only facilitate analysis across monocot species but also provide a unique ability to compare the efficiency and stability of gene editing between two species.

**Fig. 1:**
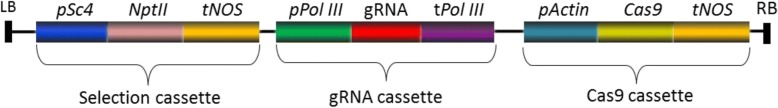
Schematic of binary plasmid T-DNA region transferred to wheat or barley. All constructs conform to this structure with differences as indicated in Table 1 for the specific *Pol III* promoters included

A total of six constructs (Table 1, Fig. 1) were used in a series of stable *Agrobacterium*-mediated transformations in wheat, with one of these, pRMH110, also used for *Agrobacterium*-mediated barley transformation. These constructs enabled analysis of the targeting efficiency within multiple and single genomes, a study of the potential for off-target effects, comparison of editing between wheat and barley and an examination of the efficiency of gene editing per se.

**Table 1:** Details of constructs used

Construct name	Wheat genome targeted	*Pol III* promoter
pRMH110	A/B/D	*TaU6*
pRMH120/123	A	*TaU6*
pRMH121	B	*OsU3*
pRMH125	D	*TaU3*
pRMH131	A/B/D and A only	*OsU3* and *TaU6*

### Gene editing targeted to three genomes

Vector pRMH110 contains a tri-genome targeted guide designed to edit all three wheat PDS homoeologues (Tables 1 and 2, Additional file 1: Figure S1). Thirty-eight transgenic T_0_ wheat lines were generated and edits identified by Sanger sequencing. Two lines were shown to contain heterozygous edits, however, while the guide was tri-genome targeted the resulting edits where only observed within single gene homoeologues. Transgenic lines GE1–2 and GE1–31 were edited in the A and B *TaPDS* homoeologues, respectively (Table 3). The nature of the edits were confirmed by cloning and Sanger sequencing multiple colonies containing the target *TaPDS* PCR amplicons. In both cases, the edit was shown to be a single base pair deletion 4 bp from the protospacer adjacent motif site (Table 3, Additional file 3: Table S2). The use of a tri-genome targeting gRNA has, therefore, resulted in an editing efficiency of 5.3% (Table 4) of single *TaPDS* homoeologues. This efficiency is comparable to those previously reported in wheat [10].

**Table 2:** Degree of similarity of individual genome guides to homeologous genes in wheat

Vector	Genome targeted	Genome	Target sequence	Mismatches (bp)
pRMH110	All genomes	gRNA	TTGTTTGCCAAGATTTTCCA	
		A	TTGTTTGCCAAGATTTTCCA	n/a
		B	TTGTTTGCCAAGATTTTCCA	n/a
		D	TTGTTTGCCAAGATTTTCCA	n/a
		Barley	TTGTTTGCCAAGATTTTCCA	n/a
pRMH120 and pRMH123	A genome	gRNA	CTGTGTATGAAGTTGTCCGG	
		A	CTGTGTATGAAGTTGTCCGG	n/a
		B	CTG**C**GTATGAAGTTGTCC**A**G	2
		D	CTGTGTATGAAGTTG**C**CC**A**G	2
pRMH121	B genome	gRNA	CTGCGTATGAAGTTGTCCAGC	
		A	CTG**T**GTATGAAGTTGTCC**G**GC	2
		B	CTGCGTATGAAGTTGTCCAGC	n/a
		D	CTG**T**GTATGAAGTTG**C**CCAGC	2
pRMH125	D genome	gRNA	GCCAGGGGAAGTCGAACTAA	
		A	GCCAAGGGA––––––––––––	11
		B	GCCAAGGGA––––––––––––	11
		D	GCCAGGGGAAGTCGAACTAA	n/a

‘-‘ absence of nucleotide, *bold* nucleotide substitution

**Table 3:** Type of edits observed in wheat T_0_ plants from different guides

Genome target	Plant ID	Copy number	Edit type^a^	Mutation detected (bp)^b^
A/B/D	GE1–2	4+	Het A	−1
	GE1–31	4+	Het B	-1
A only	GE6–4	4+	Bi-allelic A	−2/−16
	GE6–8	4+	Bi-allelic A	+ 1/−2
	GE6–22	4+	Bi-allelic A	Complex^c^
	GE6–30	3	Het A	-1
	GE6–31	4+	Het A	+ 1
	GE6–53	4	Bi-allelic A	+ 1/+ 1
D only	GE7–5	3	Het D	-1
	GE7–10	4+	Het D	−2
	GE7–21	4+	Het D	+ 1
	GE7–28	4+	Het D	+ 1
A only	GE8–1	4+	Het A	n/a
	GE8–15	4+	Het A	−2
	GE8–30	2	Het A	-2
	GE8–31	1	Het A	+ 1
	GE8–36	4+	Het A	+ 1
A/D only Co-transformation	GE11–13	4+	Het D	−12
	GE11–23	4+	Het A	+ 1
	GE11–24	4+	Het D	−15
	GE12–1	2	Het A	+ 2
	GE12–14	4+	Het D	−3
	GE13–2	3	D	n/a
	GE13–8	4+	Het D	+ 1
	GE13–28	4+	Het D	−16
	GE13–36	4+	Het D	−12
	GE13–38	4+	Het D	+ 1
	GE13–42	4	Het A	−1
	GE13–50	1	Bi-allelic D	−12/−2
	GE13–51	4+	Het D	−7
A/B/D and A double vector	GE15–1	1	Het A	−2
	GE15–16	4+	Het A	+ 1
	GE15–22	3	A	Chimeric
	GE15–28	4+	Het A	+ 1

^a^*Het* heterozygous, *Bi-allelic* two different edits on the same genome copy; letter indicates edited genome,

^b^−/+ = deletion or insertion and number of base pairs involved, *n/a* data not available

^c^Complex edit which contains both inserts and deletions. For sequences see Additional file 3: Table S2.

**Table 4:** Transformation and editing efficiencies

Experiment	Construct	Genome target	No. of inoculated embryos	No. of transgenic lines	No. of edited lines (editing efficiency, %)	Embryo to edit efficiency
GE1	pRMH110	A/B/D	248	38	2 (5.3)	0.8
GE2/4	pRMH121	B only	157	28	0 (0)	–
GE6	pRMH120	A only	227	52	6 (11.5)	2.6
GE7	pRMH125	D only	213	35	4 (11.4)	1.9
GE8	pRMH123	A only	210	30	5 (16.7)	2.4
GE11/12/13^b^	pRMH120 / pRMH125	A and D	432	73	13 (17.8)	3
GE15/16	pRMH131	A and all three double	223	27	4 (14.8)	1.8
Hv6	pRMH110	N/A	66	40	6 (15^a^)	9

*GE* Wheat experiments, *Hv* Barley experiment

^a^High levels of somatic editing were observed making the true edit efficiency unclear

^b^Co-transformation of two different *Agrobacterium* strains containing different constructs

### Individual genome targeting and off-target edits

The use of CRISPR gene editing on individual gene homoeologues is dependent on the identification of target sequences with a sufficient level of difference. The level of similarity between wheat homoeologues is often high, resulting in limited options for homoeologue specific guide design. To further analyse this, we produced constructs designed to target the individual genomes of wheat (Table 1); the guides designed are identical to the targeted genome but have differing degrees of similarity to the other two genomes (Table 2). This not only provided a starting point for the evaluation of the degree of similarity possible without increasing the levels of off-target effects, but also provided a means by which the functionality of the Pol III promoter options could be tested in wheat.

Only limited options were available to enable targeting of the B genome homoeologue, and in accordance with the literature, where additional nucleotides have been added to the start of the gRNA without deleterious effect [15–17], an additional adenine residue was added to enable this to be transcribed from the *OsU3* promoter. Successful editing was achieved for two of the three guides; vector pRMH125 targeting the D genome homeologue and vectors pRMH120 and pRMH123 targeting the A homeologue. These produced editing efficiencies of 11.4, 11.5 and 16.7% respectively (Table 4), a higher editing efficiency than that previously published [7, 8].

The use of individual genome targeting has also enabled the potential for off-targets to be studied using the other TaPDS homoeologues. The D genome guide has a very high specificity with 11 bp at the 3′ end of the guide absent in the sequence of both the A and B genome homeologues. While the A genome targeted guide has only 2 nucleotides differing from the B and D homoeologues, sequencing showed that no off-target edits were produced in non-targeted homoeologues.

No edited plants were obtained with the B genome targeted pRMH121 vector, suggesting either the *OsU3* guide scaffold does not work as efficiently in wheat in directing the Cas9 to the target sequence, the gRNA design was not optimal or that the additional A nucleotide reduced the efficiency of the editing by weakening the stability of the guide.

Comparing the efficiencies of the successful single genome targeting experiments (Table 4), it is possible to see that the editing is higher than that produced by the tri-genome targeted guide and that the vectors have similar efficiencies regardless of the stringency of the guide to the genome or the promoter used, with the exception of those containing *OsU3*.

Using our high efficiency wheat transformation method, the single genome targeted guides produce an inoculated embryo to edited plant efficiency of 1.9–3%.

### Co-transformation of single genome guides

Given the higher efficiency achieved with single genome guides compared with tri-genome guides, a possible method by which multiple genome editing could be achieved would be to use two *Agrobacterium* cultures, each containing a separate construct, in a co-transformation experiment. Constructs pRMH120 (A genome) and pRMH125 (D genome) were co-transformed using a 50:50 ratio; 73 transformed lines were obtained with an overall editing efficiency of 17.8%, the highest seen across all the experiments. Identification of which T-DNA was present within each of the plants showed that the pRMH120 T-DNA (A genome guide) was present in 59% of plants while the pRMH125 T-DNA (D genome guide) was found in 74% of plants. Taking the T-DNA presence into account it is possible to determine that not only was the gRNA for the D genome present in more lines, but also that the efficiency of editing was substantially increased with 26.6% of plants edited, compared to the 11.4% achieved when transformed individually (Table 5). In contrast, the efficiency of the A genome specific gRNA was reduced from 11.5 to 5.3% in the co-transformation experiment (Table 5). In 33% of lines, both T-DNA copies were present and an equal ratio of editing efficiency was observed for both homoeologues. Noticeably, no lines with both genomes edited were produced in these experiments.

**Table 5:** Percentage of transgenic wheat plants edited in co-transformation experiment compared to when transformed alone

Genome target	Vector	Transformation efficiency (%)	
		Single constructs	Co-transformation
A	pRMH120	11.5	5.3
D	pRMH125	11.4	26.6

### Larger deletion evaluation

Finally, a construct was designed to evaluate the possibility of creating larger deletions in wheat, which has been demonstrated using CRISPR/Cas9 in both rice and maize [3, 18]. Construct pRMH131 contained Cas9 plus two guide sequences; the first, the A genome specific guide expressed from the *TaU6* promoter, as used in previous experiments, and the second, a new tri-genome guide expressed from the *OsU3* promoter. This, we anticipated, would enable us to create standard edits within the B and D genome and had the potential for larger deletions in the A genome.

Sequencing the target gene homoeologues from lines produced with pRMH131 showed a 14.8% efficiency of A genome editing, in agreement with the previous experiments using this guide. However, no edits were identified in any of the genomes at the multi-genome target site, as with the previous experiments using the *OsU3* promoter.

### Type of edits

Sequencing the target gene homoeologues in each T_0_ plant enabled us to further examine the type of edit produced in our system (Table 3). Across all experiments, 50% of mutations are single base pair insertion or deletion edits, as seen in other species [19] and, overall, 58.8% of all edits are deletions. It would also appear that guide design may have an impact on the type of edit being created as the A genome guide from pRMH120 and pRMH123 produced 22.2% of bi-allelic edits whereas the D genome guide from pRMH125 produced just 8.3%.

### Stable inheritance of the gene edits

The stable inheritance of edits *in planta* is a much discussed topic, with reports of unpredictable non-Mendelian inheritance in subsequent generations, and the general conclusion is that some edits detected are somatic in nature [4, 5, 20]. Reports indicate that additional editing occurs in subsequent generations and that stable edits are achieved only after the removal of the transgene [6, 18, 21]. Here, we report the first published work, to date, showing stable Mendelian heritability of edits in transformed wheat lines over multiple generations (Table 3).

To determine both the stability of the edit and the ongoing activity of the CRISPR/Cas9, five edited plus seven non-edited lines were progressed to the T_1_ generation. None of the lines exhibited any additional editing in the next generation, indicating that editing occurred early and that despite the presence of a functional Cas9, additional edits were not produced. This is in contrast to previously published results in wheat [6]. Analysis was carried out on the T_1_ progeny of the five selected edited plants, four plants heterozygously edited in the T_0_ (GE1–2, GE1–31, GE7–5 and GE8–30) while the fifth plant (GE13.50) contains biallelic edits. In all cases the observed T_0_ edits were seen to segregate in a Mendelian fashion with 1:2:1 ratio confirmed using a χ^2^ test (Table 6).

**Table 6:** Segregation of edit in T_1_ plants. Number of plants of each genotype is indicated

T_0_ line	Homozygous edit	Heterozygous edit	WT	χ^2^ *P* value
GE1–2	8	8	3	0.212
GE1–31	6	14	8	0.867
GE7–5	6	14	9	0.721
GE8–30	11	27	5	0.106
T_0_ line	Homozygous allele 1	Bi-allelic	Homozygous allele 2	χ^2^ *P* value
GE13–50	7	13	3	0.410

The selected lines cover a range of T-DNA copy number (Table 3), GE1–2 and GE1–31 were selected for further generation analysis despite the high 4+ copy due to the presence of the tri-genome targeting guide within these lines. Given the known activity of the Cas9 in the T_0_ generation it was anticipated that these lines might produce additional edits within the non-edited genomes in subsequent generations. However, no additional edits were seen in the T_1_ generation despite the continued presence of the T-DNA containing the Cas9-gRNA. This is in contrast to observations within rice [5, 12, 19], wheat [6] and other species [4, 22] where new edits were introduced.

Four T_1_ lines, GE1–2-6, GE1–31-5, GE7.5–1 and GE7–5 − 12, with confirmed homozygous edits were selected and T_2_ embryos rescued. As anticipated, the expected edit was seen in 100% of the T_2_ lines, and again no new edits were identified. Analysis of T-DNA insert was carried out using both an *nptII* copy number assay and a Cas9 absence/presence PCR and the results show the expected presence of both full length and partial T-DNA inserts. However, prudent selection of lines at both the T_0_ and T_1_ generation can enable rapid progression to homozygous edited, Cas9 free plants (see Additional file 4: Table S3).

GE7–5-1 was selected in the T_1_ generation using *nptII* copy number assay with zero copies detected. A single partial T-DNA insertion bearing Cas9 segregated in this line with the expected 1:2:1 ratio, resulting in 25% Cas9/T-DNA free edited T_2_ lines identified (Additional file 4: Table S3). GE7–5-12 was identified as 4 copy, and thus, the segregation of the T-DNA would only be expected to produce a limited number of null segregants, however we analysed 23 lines and successfully identified a single null segregating plant (Additional file 4: Table S3). The remaining two lines GE1–2-6 and GE1–31-5 both showed 4+ copies of *nptII* and in the limited seed produced from a single ears, with 16 plants and 24 plants germinated respectively, it was not possible to identify Cas9 free lines. T_0_ lines with lower *nptII* copy number such as GE8–30 which contains two T-DNA copies plus a partial T-DNA, can be rapidly progressed through the T_1_ generation in order to select for the Cas9 free plants in the T_2_ generation (Additional file 4: Table S3). Even plants indicating a higher *nptII* copy number, such as GE13–50, are able to produce lower copy number lines within the T_1_ generation, if the edit is considered to be of greater importance (Additional file 4: Table S3).

Given the lack of additional edits observed in subsequent generations it was postulated that a callus stage might be a requirement for the activation of the CRISPR/Cas9 system and the production of edits. To test this hypothesis, T_2_ immature embryos from a homozygous edited GE1 line were used to generate 251 plantlets via callus induction and subsequent regeneration. Line GE1–31-5 was homozygous for a B genome edit created using a tri-genome targeted guide, and also retained a 4+ *nptII* copy number for the Cas9 T-DNA. Given the presence of a tri-genome targeting guide, additional edits within non-edited genomes might be anticipated if the system is reactivated via a callus induction stage. Our data indicates that this is not the case as while the B genome edit was fully retained, no additional genomic edits were observed.

### Comparison of gene editing in barley

**Fig. 2:**
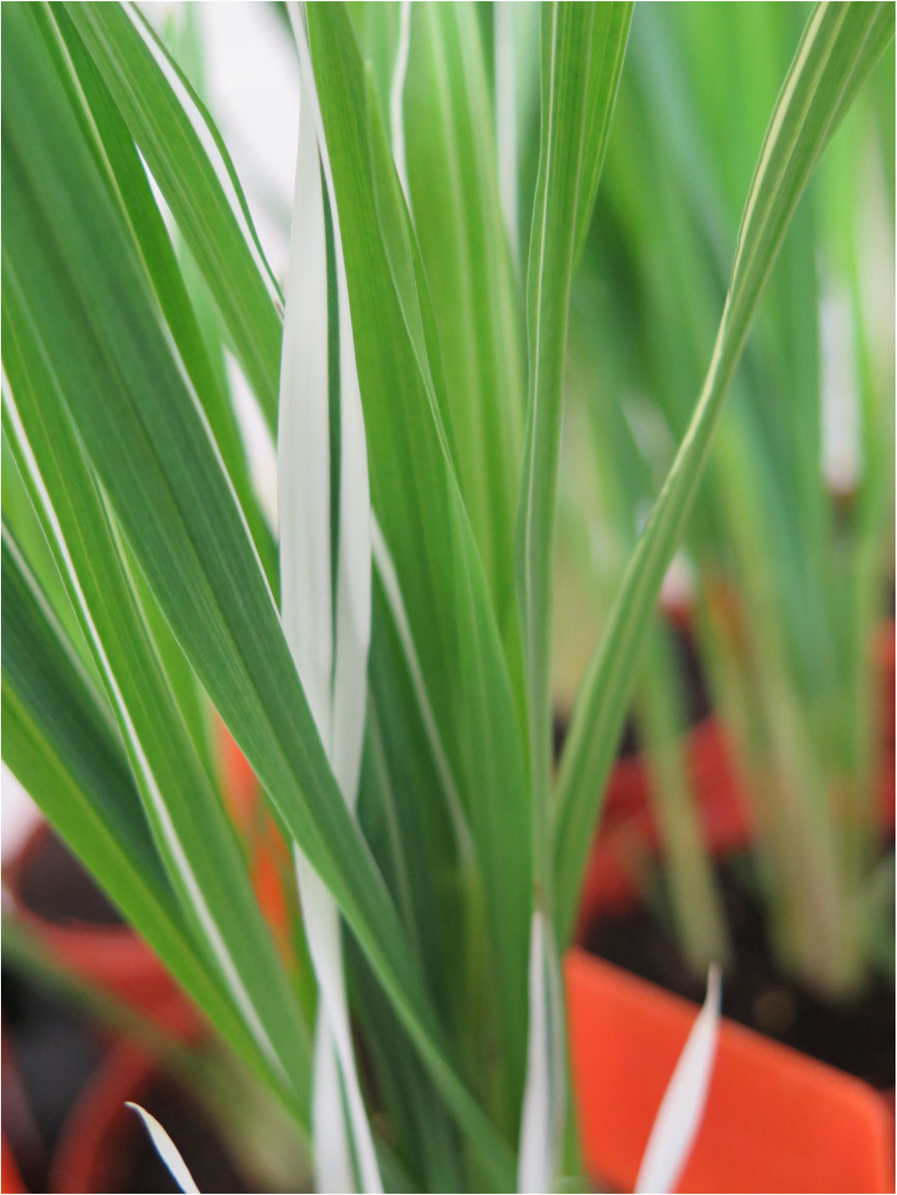
Transgenic barley plant exhibiting chimaeric photobleaching phenotype

The tri-genome guide contained within pRMH110 is also able to target the barley *HvPDS* gene, with a 100% identity for the guide. Forty stable independent transformed barley lines where examined, of which six demonstrated editing. This gives an efficiency of editing for the single *HvPDS* gene in diploid barley of 15%, compared to 5% efficiency for the same tri-genome targeting guide in hexaploid wheat. The cloning and sequencing of the potentially edited barley lines showed larger deletions of up to 350 bp, and also demonstrated the presence of somatic editing. In contrast to wheat, cloning and sequencing the barley T_0_ amplicons showed multiple genotypes to be present within a single plant in 5 of the 6 lines, with up to 4 different genotypes within a single leaf sample. This is borne out in the phenotype exhibited by the plants, which showed chimeric photobleaching (Fig. 2). A closer examination of the chimeric regions, using DNA from striped, white and completely green leaf areas, showed that these corresponded to regions where heterozygous and homozygous edits or WT sequences were detected. Re-sampling of plants 44 days later produced a different series of edits suggesting that the edit obtained is specific to the section of leaf rather than true plant genotype (data not shown). Previous published work in barley also suggests that edits could be somatic, which is in line with our results [4]. This is in contrast to our results in wheat, where chimaerism was observed in only one of the 34 edited lines (GE15–22) which was detected by sequencing the homeologue target region, and identifying four sequence variants including the WT (Table 3).

## Discussion

The results presented here show that our *Agrobacterium*-mediated transformation system in wheat is able to produce an editing efficiency equivalent to the previously published efficiencies using other transformation methods [7, 8]. Indeed, this efficiency is within the range commonly reported for standard wheat transformation *per se* [23]. This demonstrates that targeted mutagenesis of individual homeologues within the wheat genome is achievable with a modest amount of effort, and without associated off-target mutations. While the editing efficiencies observed in wheat were within the ranges anticipated, no edits were obtained using the OsU3 promoter. The increased ability of the gRNAs expressed from the *TaU3* promoter to introduce edits compared with *OsU3* has previously been noted in maize protoplasts [16] and the lack of edits using *OsU3* with either of the two guides, single or tri-genome targeted, would suggest that it is not acting efficiently enough in our wheat experiments to produce edits.

Across all the experiments carried out, edits were produced in each of the three *TaPDS* homoeologues such that the resultant protein would be truncated, however, no phenotype was observed indicating that the other two genomes can compensate for the loss of a single genome copy. Our targeting of the individual wheat genomes has enabled a closer examination of the potential for generation of off-target edits and our results indicate that even with limited nucleotide differences between the genome sequences, no off-target editing occurred within the non-targeted homeologues. This result was unexpected given previous reports found that off-target effects were seen between the homeologues of wheat genes [10]. Our results indicate that high levels of similarity can be used within this system without resulting in off-target edits, however, a more detailed examination of multiple guides would be required to determine whether the position of mismatches within the guide results in increased off-target editing.

The use of co-transformation for editing has demonstrated that while the levels of editing efficiency observed might not produce multiple homeologue knockouts in a single plant, it would provide an excellent means by which different homeologues or genes could be knocked out in wheat via a single transformation experiment. This would not only reduce the time required for multiple plant transformation experiments but also reduce the total number of T_0_ plants required, and thus, the overall amount of molecular analysis and, therefore, greatly reduce costs.

A single construct containing both guides, rather than using co-transformation with separate guides, would ensure that the editing machinery is being introduced into the same cell and increase the likelihood of a line edited in both genome copies being produced. Conversely, the lower ratio of Cas9 protein per gRNA compared with the single vector system could potentially reduce efficiency. Cas9 expression could be elevated with the use of a stronger promoter, such as maize ubiquitin, however whether this may also have deleterious effects such as ongoing editing, or higher levels of somatic editing, remains to be determined.

In our wheat experiments, the predominant type of edit introduced was a single nucleotide insertion or deletion as observed in other species [19], however, the percentage of bi-allelic edits was lower than that observed in rice [5, 20]. This may suggest that a more stringent DNA break repair system is present in wheat. Inter-species differences have also been demonstrated by our observation that the same construct produces different patterns of editing between the two closely related species, wheat and barley. It would appear that in wheat the editing occurs at an early stage and is stable, whereas in barley editing continues to occur (including at somatic level) and is less stable. These observations would indicate, therefore, that for optimum results, both construct components and subsequent analysis should be tailored to the individual species.

Our experiments on inheritance of edits across multiple generations demonstrates the necessity for the early establishment of T-DNA copy number for lines, and that careful selection of lines not only for edit, but also T-DNA copy number, is required to rapidly progress to the successful identification of edited T-DNA free lines. Using embryo rescue it was therefore possible to achieve stable edited Cas9 free T_2_ wheat plants from the initiation of plant transformation in only 36 weeks. This rapid progression to stable edited transgene free T_2_ lines is only made possible as a result of the Mendelian inheritance of the edit, even in the presence of the Cas9 in the T_1_ generation. Our results appear to contrast with previously published work in monocots [4, 11] where edits are of a more complex bi-allelic nature, including chimeric, and T_1_ progeny do not follow the expected Mendelian segregation ratios in the presence of Cas9 or where additional editing is observed [6]. It is unclear at present the reason for this contrasting result but we would postulate that the vector design is the most likely source of this difference.

## Conclusions

Our work shows that it is possible to obtain efficient gene editing in wheat using *Agrobacterium*-mediated stable transformation of CRISPR/Cas9 constructs; our embryo to edit efficiency is broadly equivalent to the efficiency of wheat transformation alone as reported by other researchers. We demonstrate that the same construct can produce different patterns of editing in two different monocot crops, showing the importance of tailoring both the construct design and the downstream analysis to the individual plant species. Within our system, edits occur early in the wheat transformation process and the edits are stable within the T_0_ plant and subsequent generations. In contrast, edits continue to occur in barley, and are often somatic in nature. Our results indicate that off-target editing has not occurred in wheat in this study, and that edits are heritable in a Mendelian fashion in presence of Cas9 from the first generation. Whether this is a consequence of the differing ploidy levels in the two species remains unclear. This work demonstrates that it is possible to edit hexaploid wheat and obtain T-DNA/Cas9 free edited plants in a relatively short space of time, without the need to screen large numbers of plants. We demonstrate that co-transformation could be used as an effective means of obtaining multiple edits through a single transformation experiment without loss of efficiency. While other published work has reported the requirement for rapid removal of Cas9 and complex bi-allelic and chimeric editing patterns with continuing editing, our system demonstrates an efficient strategy by which multiple “easy to breed” edits can be obtained from a single transformation experiment. The lack of additional editing in subsequent generations and Mendelian segregation observed, even in the continued presence of Cas9, provides the ability to rapidly progress those lines through multiple generations to obtain T-DNA clean, stable homozygous edited lines for phenotypic evaluation.

## Methods

### Design of vectors

The *Cas9* gene sequence [7], including the nuclear localisation signals, was codon optimised for wheat and synthesised (Genscript, Piscataway, NJ, USA), and recombined into vector pSc4ActR1R2 [24] using a Gateway Clonase II kit (Thermo Fisher Scientific Inc.). In the resulting vector, pRMH088, the Cas9 gene is expressed from the rice *Actin* promoter [25] and transcripts terminated by the nopaline synthase terminator (*tNOS*) from *Agrobacterium tumefaciens.* The vector which also contains the *nptII* (*neomycin phosphotransferase II*) gene expressed from the *subterranean clover stunt virus* 4 promoter [26] with the *Arabidopsis thaliana FAD2* intron [27] and transcripts were terminated by tNOS.

Basic gRNA vectors were designed containing one of three *Pol III* polymerase promoter and terminators, *TaU3* [18], *TaU6* from wheat or *OsU3* [7] from rice and synthesised in the pUC57 backbone (Genscript, Piscataway, NJ, USA). PDS target guide sequences were inserted into the vectors using annealed primer pairs ligated into unique restriction sites. Each gRNA cassette was then cloned into pRMH088 using a second set of unique restriction sites.

### Gene identification

The wheat *phytoene desaturase* (*TaPDS*) gene was identified using the rice PDS gene (GenBank accession AF049356) as a query for a BLAST [28] analysis of the wheat genome reference sequence, IWGSCv1 wheat genome [29]. Gene predictions for all three homoeologues were produced by alignment of each genomic sequence to identified wheat expressed sequence tag (EST) sequences using est2genome [30]. The first five exons and four introns of *TaPDS* were amplified from the US spring variety Fielder (USDA, ARS) using TaPDS genomic sequence primers (Additional file 1: Figure S1 and Additional file 2: Table S1) and Phusion Hotstart II polymerase (Thermo Fisher Scientific Inc.). Amplicons were cloned into the pGEM-T Easy vector system (Promega), a number of colonies were sequenced and the varietal sequence for the three homoeologues confirmed.

Primers were designed to genome specific SNPs identified from *TaPDS* sequences (Additional file 2: Table S1) and PCR amplicons tested for genome-specificity using the Chinese Spring nulli-tetrasomic lines [31]. PCR conditions with primers at a final concentration of 1 mM, 0.4 U FastStart *Taq* DNA polymerase (Sigma-Aldrich) and using the provided standard 10× reaction buffer with MgCl_2_ to a final concentration of 2 mM and dNTPs to a final concentration of 1 mM. PCR conditions were all as follows: (95 °C 4mins [95 °C 30s, 64 °C 30s, 71 °C 1 min] × 40, 72 °C 10mins).

Barley *HvPDS* genomic sequence was identified using the *TaPDS* homoeologues in a BLAST [28] query of the barley genome assembly 082214v1, in the variety Morex [32], and aligned with the wheat sequences using AlignX (Thermo Fisher Scientific Inc.). The identified region of similarity was amplified from variety Golden Promise and examined in transgenics with the primers listed in Additional file 2: Table S1 using the PCR conditions above with annealing temperature of 54 °C.

### Design of gRNA

Target sequences were selected within the first two exons of the gene using the Emboss tool DREG [30], with the sequence specification of G(N)_21_GG or A(N)_21_GG. Gene specificity was confirmed using a BLAST of the target sequence to the IWGSCv1 wheat genome. Genome specificity and promoter type were used to select the gRNA sequence for each construct.

### Plant growth, tissue culture and transformation

Completed constructs were verified by restriction digest and sequencing before being electro-transformed into *Agrobacterium tumefaciens* strain AGL1 [33]. Plasmids were isolated from *Agrobacterium* colonies selected on media containing kanamycin (50 μg/ml) and rifampicin (50 μg/ml) then verified by restriction digest prior to use in wheat or barley transformation experiments [34].

Fielder wheat stock plants (USDA, ARS) were grown in M2 compost plus 5 g/l slow release fertilizer (Osmacote Exact 15:9:9) in a controlled environment chamber (Conviron) at 20 °C day/15 °C night with a 16 h photoperiod (approximately 400 μE m^− 2^ s^− 1^). Immature seeds were harvested for transformation experiments at 14–20 days post-anthesis (dpa). Wheat embryos were isolated and then co-cultivated with *Agrobacterium tumefaciens* for 2 days in the dark [35]. The embryonic axis removal and subsequent tissue culture steps were performed as described by Risacher et al. [36]. Individual plantlets were transferred to Jiffy-7 pellets (LBS Horticulture) and hardened off, then potted up into 9 cm plant pots and grown on to flowering in controlled environment chambers, as above.

Embryo rescue was performed by isolating embryos (12–18 dpa) aseptically, followed by culture, embryo axis uppermost, on MRMZ2 medium (MRM medium [36] supplemented with 2 mg/l zeatin in place of kinetin). After approximately 5–7 days on MRMZ2, germinated shoots were transferred to 40-well tray inserts containing Levingtons M2 compost with added Osmocote exact slow release fertilizer (LBS horticulture), and grown on in plant growth chambers, as above. For callus induction experiments, immature embryos (14–20 dpa) were isolated aseptically and placed, scutellum uppermost, onto W425G medium [36]. After 3 days, axes were excised and the scutella transferred to fresh W425G. Following a further transfer to W425G, with a total of 6 weeks on selective callus induction medium, calli were transferred to MRMZ2-25G medium for regeneration, followed by transfer of shoots into Beatson jars containing MS20 medium. After 4 weeks, plantlets were sampled for analysis.

*Agrobacterium tumefaciens*-mediated transformation of barley variety Golden Promise (JIC, UK) was carried out, using immature embryos, as per published protocols [37].

### Plant DNA extraction

Chinese Spring nulli-tetrasomic lines [31] were grown under poly-tunnel conditions, leaves sampled from individual lines, and genomic DNA extracted using a modified Tanksley extraction method [38].

DNA from transgenic wheat and barley lines was extracted using crude DNA extraction buffer (200 mM Tris pH 7.5, 250 mM NaCl, 25 mM EDTA, 0.5% SDS), incubated at 65 °C for 1 h then centrifuged at 6000 g for 10 min. The DNA was precipitated by addition of 400 μl isopropanol to the supernatant followed by centrifugation, as previously. DNA pellets were resuspended in 100 μl TE, incubated at 65 °C for 5 min and centrifuged at 6000 g for 5 min. DNA was diluted 1:3 in sterile H_2_O prior to use in all assays.

### Plant analysis

T-DNA copy number was determined using a TaqMan relative quantification (ΔΔCT) assay comparing the relative values of an *nptII* amplicon to an amplicon of the single copy wheat gene *GaMyb* within a multiplexed reaction and normalised to a single copy control [39]. All reactions are carried out using two replicates per plant line. Primers and probes (Additional file 2: Table S1) were used at a final concentration of 10 μM in 10 μl reactions with ABsolute Blue qPCR ROX mix (Thermo Fisher Scientific Inc.) using the standard run conditions for the ABI 7900 HT (Thermo Fisher Scientific Inc.).

*Cas9* presence/absence PCR was carried out using the standard FastStart *Taq* DNA mix and conditions (as above) with an annealing temperature of 57 °C and extension time of 1 min 30s.

For determination of edits, genome specific PCRs for all three homoeologous genes were conducted on each transgenic plant. Amplicons were purified using an Exo-SAP reaction (Thermo Fisher Scientific Inc.) and Sanger sequencing carried out using ABI Big Dye Mix v3.1 (Thermo Fisher Scientific Inc.) with the provided protocol, and run on an ABI 3730 instrument (Thermo Fisher Scientific Inc.).

Where the plant line showed an edit based on an initial sequence chromatogram, the amplicon was cloned into pGEM-T Easy (Promega) and 5–20 colonies for each amplicon sequenced as previously. Traces were examined using a combination of Contig Express and AlignX (Thermo Fisher Scientific Inc.).

## Additional files

Additional file 1: **Figure S1.** Sequence alignment of TaPDS from wheat variety Fielder and HvPDS from barley variety Golden Promise with guide sites indicated. (DOCX 149 kb)

Additional file 2: **Table S1.** Sequences of primers used. (XLSX 10 kb)

Additional file 3: **Table S2.** Edits identified in T_0_ plants. (DOCX 24 kb)

Additional file 4: **Table S3.** Segregation of Cas9 and edits within the T_1_ and T_2_ plants. (XLSX 19 kb)

## Abbreviations

CRISPR: Clustered regularly interspaced short palindromic repeats
EST: Expressed sequence tag
gRNA: Guide RNA
*nptII*: Neomycin phosphotransferase
PDS: Phytoene desaturase
Pol III: Polymerase III
RNAi: RNA interference
SNPs: Single nucleotide polymorphisms
tNOS: Nopaline synthase terminator
VIGS: Virus induced gene silencing
