# Supplementary material for: Efficient generation of stable, heritable gene edits in wheat using CRISPR/Cas9
Source: BMC Plant Biol. 2018 Oct 3;18:215. doi: 10.1186/s12870-018-1433-z (PMC6171145; doi:10.1186/s12870-018-1433-z)
Supplement: Supplementary file 1 — Figure S1. Sequence alignment of TaPDS from wheat variety Fielder and HvPDS from barley variety Golden Promise with guide sites indicated. (DOCX 149 kb) [file 12870_2018_1433_MOESM1_ESM.docx]

Figure S1. Sequence alignment of TaPDS from wheat variety Fielder and HvPDS from barley variety Golden Promise with guide sites indicated.


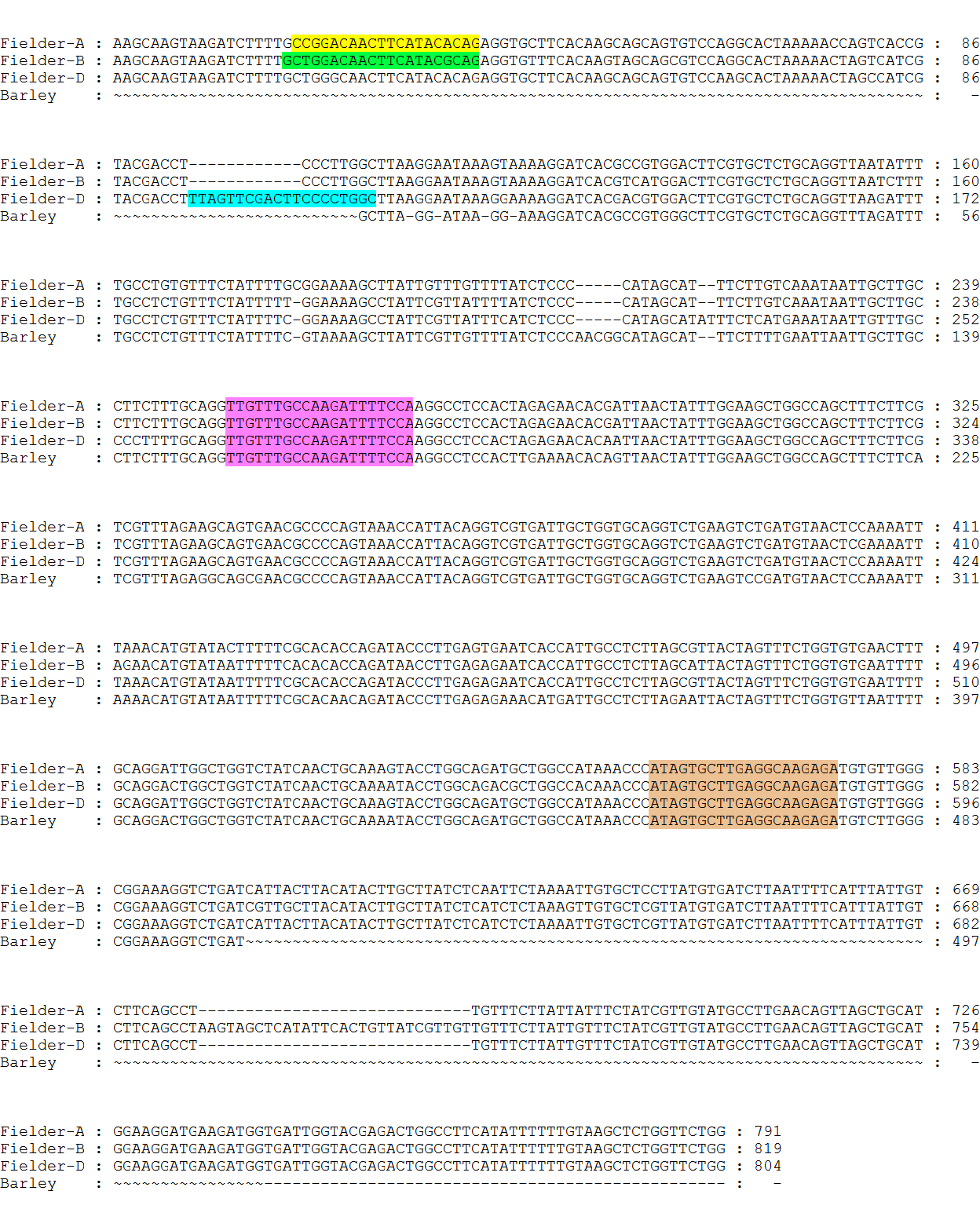


Guide regions for vectors are coloured as follows:-

Yellow, TaU6 A genome guide (pRMH120, pRMH123 and pRMH131); Green,OsU3 B genome guide (pRMH121); Blue, TaU3 D genome guide (pRMH125), Pink, TaU6 tri-genome (pRMH110); Orange, OsU3 tri-genome (pRMH131)
