# Supplementary material for: Efficient generation of stable, heritable gene edits in wheat using CRISPR/Cas9
Source: BMC Plant Biol. 2018 Oct 3;18:215. doi: 10.1186/s12870-018-1433-z (PMC6171145; doi:10.1186/s12870-018-1433-z)
Supplement: Supplementary file 3 — Table S2. Edits identified in T0 plants. (DOCX 24 kb) [file 12870_2018_1433_MOESM3_ESM.docx]

| **Plant ID** | **Edit type** | **Mutation detected** |
| --- | --- | --- |
| **GE1** | **WT genome** | TTGTTTGCCAAGATTTTCCAAGGTGCTT |
| GE1-2 | Het A allele | TTGTTTGCCAAGATT**–**TCCAAGGTGCTT |
| GE1-31 | Het B allele | TTGTTTGCCAAGATT**–**TCCAAGGTGCTT |
|  |  |  |
|  |  |  |
| **GE6** | **WT A genome** | CCGGACAACTTCATACA---C-------------------------------AGAGGTGCTT |
| GE6-4 | Bi-allelic allele 1 | CCGGACAACTTCATA**--**---C-------------------------------AGAGGTGCTT |
| GE6-4 | Bi-allelic allele 2 | CCGGACAACT**-------**---**-**-------------------------------**--------**TT |
| GE6-8 | Bi-allelic allele 1 | CCGGACAACTTCATA**--**---C-------------------------------AGAGGTGCTT |
| GE6-8 | Bi-allelic allele 2 | CCGGACAACTTCATACA**A**--C-------------------------------AGAGGTGCTT |
| GE6-22 | Bi-allelic allele 1 | CCGGA**------**CATA**--**---**-**----------------------------**GCT**AGAGGTGCTT |
| GE6-22 | Bi-allelic allele 2 | CCGGACAACTTCATA**GTTTGCTGAGCAAAAGATCTCACACTGCTTGAGCAAA**AGAGGTGCTT |
| GE6-30 | Het allele | CCGGACAACTTCATAC**-**---C-------------------------------AGAGGTGCTT |
| GE6-31 | Het allele | CCGGACAACTTCATACA**A**--C-------------------------------AGAGGTGCTT |
| GE6-53 | Bi-allelic allele 1 | CCGGACAACTTCATACA**T**--C-------------------------------AGAGGTGCTT |
| GE6-53 | Bi-allelic allele 2 | CCGGACAACTTCATACA**A**—-C-------------------------------AGAGGTGCTT |
|  |  |  |
|  |  |  |
| **GE7** | **WT D genome** | GCCAGGGGAAGTCGAAC-TAAAGG |
| GE7-5 | Het allele | GCCAGGGGAAGTCGAA**-**-TAAAGG |
| GE7-10 | Het allele | GCCAGGGGAAGTCGA**--**-TAAAGG |
| GE7-21 | Het allele | GCCAGGGGAAGTCGAAC**A**TAAAGG |
| GE7-28 | Het allele | GCCAGGGGAAGTCGAAC**C**TAAAGG |
|  |  |  |
|  |  |  |
| **GE8** | **WT A genome** | CCGGACAACTTCATACA-CAGAGGTGCTT |
| GE8-15 | Het allele | CCGGACAACTTCAT**--**A-CAGAGGTGCTT |
| GE8-30 | Het allele | CCGGACAACTTCAT**--**A-CAGAGGTGCTT |
| GE8-31 | Het allele | CCGGACAACTTCATACA**A**CAGAGGTGCTT |
| GE8-36 | Het allele | CCGGACAACTTCATACA**A**CAGAGGTGCTT |
|  |  |  |

**Additional File 3:** **Table S2.** Edits identified in T_0_ plants.

| **GE11/12/13** | **WT A genome** | CCGGACAACTTCATACA--CAGAGGTGCTT |
| --- | --- | --- |
| GE11-23 | Het allele | CCGGACAACTTCATACA**T**-CAGAGGTGCTT |
| GE12-1 | Het allele | CCGGACAACTTCATACA**CA**CAGAGGTGCTT |
| GE13-42 | Het allele | CCGGACAACTTCATACA-**A**CAGAGGTGCTT |
|  |  |  |
|  |  |  |
| **GE11/12/13** | **WT D genome** | AAGCCAGGGGAAGTCGAAC-TAAAGGTCGTACGA |
| GE11-13 | Het allele | AAGCCAGGGGAAG**------**-**------**TCGTACGA |
| GE11-24 | Het allele | AAGCCAGGGGAA**-------**-**--------**GTACGA |
| GE12-14 | Het allele | AAGCCAGGGGAAGTCG**---**-TAAAGGTCGTACGA |
| GE13-8 | Het allele | AAGCCAGGGGAAGTCGAAC**C**TAAAGGTCGTACGA |
| GE13-28 | Het allele | AAGCCAGGGGAAGT**-----**-**-----------**CGA |
| GE13-36 | Het allele | AAGCCAGGGGAA**-------**-**-----**GTCGTACGA |
| GE13-38 | Het allele | AAGCCAGGGGAAGTCGAAC**A**TAAAGGTCGTACGA |
| GE13-50 | Bi-allelic allele 1 | AAGCCAGGGGAAGTCGAA**-**-**-------**CGTACGA |
| GE13-50 | Bi-allelic allele 2 | AAGCCAGGGGAAGTCGA**--**-TAAAGGTCGTACGA |
| GE13-51 | Het allele | AAGCCAGGGG**-------**AC-TAAAGGTCGTACGA |
|  |  |  |
|  |  |  |
| **GE15** | **WT A genome** | CCGGACAACTTCATACA-CAGAGGTGCTT |
| GE15-1 | Het allele | CCGGACAACTTCAT**--**A-CAGAGGTGCTT |
| GE15-16 | Het allele | CCGGACAACTTCATACA**T**CAGAGGTGCTT |
| GE15-28 | Het allele | CCGGACAACTTCATACA**T**CAGAGGTGCTT |

Nucleotides in red indicate PAM site; purple indicate genomic sequence outside of guide region, green indicates change from the wild type sequence
